# Supplementary material for: Phase of firing coding of learning variables across the fronto-striatal network during feature-based learning
Source: Nat Commun. 2020 Sep 16;11:4669. doi: 10.1038/s41467-020-18435-3 (PMC7495418; doi:10.1038/s41467-020-18435-3)
Supplement: Supplementary file 1 — Supplementary Information [file 41467_2020_18435_MOESM1_ESM.pdf]

Supplementary Information for:

Phase of firing coding of learning variables across the fronto-striatal network during feature-based learning

Benjamin Voloh, Mariann Oemisch, Thilo Womelsdorf

Supplementary Fig.'s S1-S11.

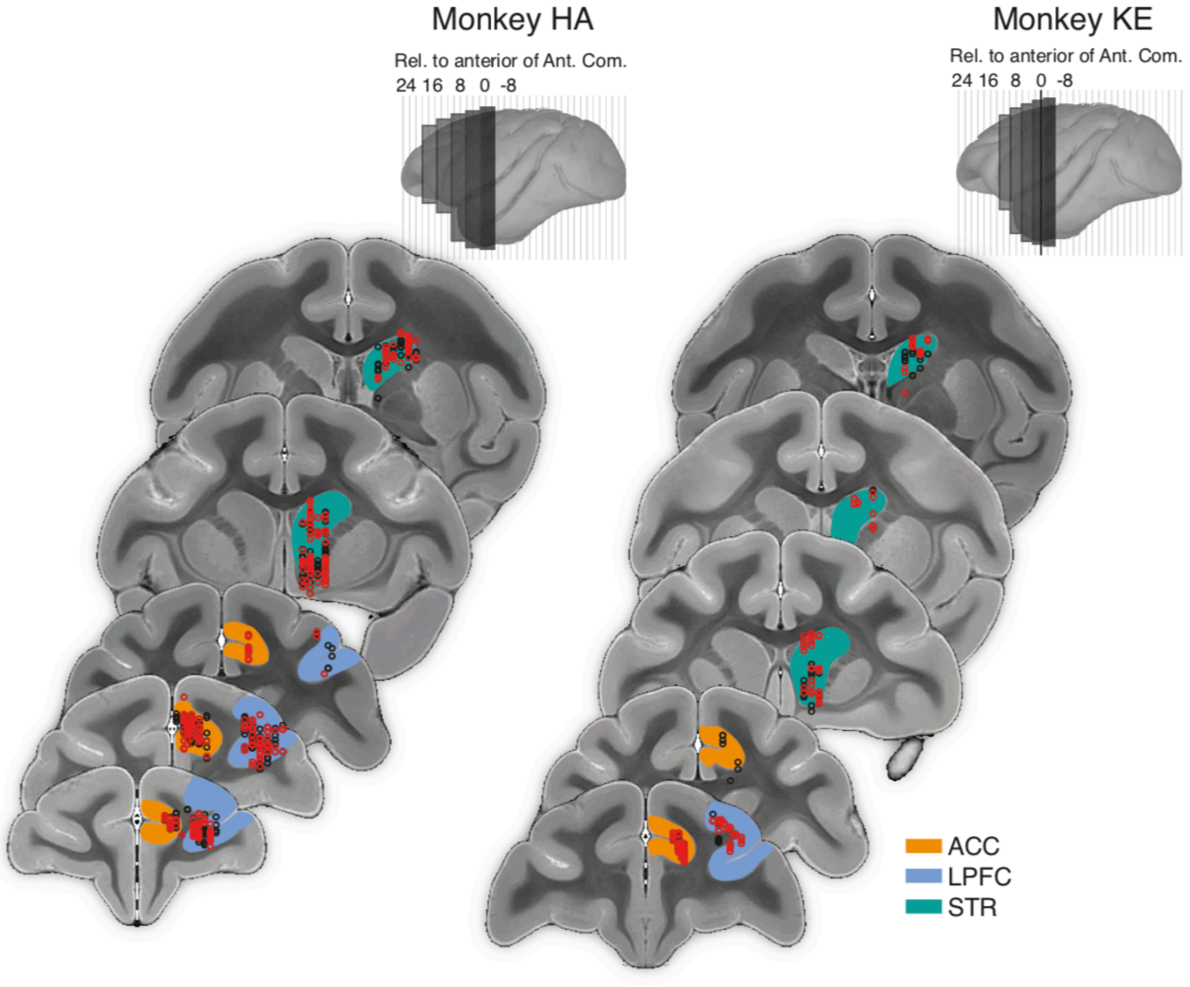

Supplementary Fig. 1. Recording sites
All recording units used in the analysis. Units were collapsed across the anterior-posterior axis into equally spaced bins for each monkey. (*top*) Slice bin limits are visualized in the top, lateral view. (*bottom*) Units depicted on a representative atlas slice. Red dots represent encoding cells, and black represents non-encoding cells. Colored outlines correspond to the ACC (orange), STR (green), and LPFC (blue). Encoding cells were broadly distributed across the fronto-striatal axis.

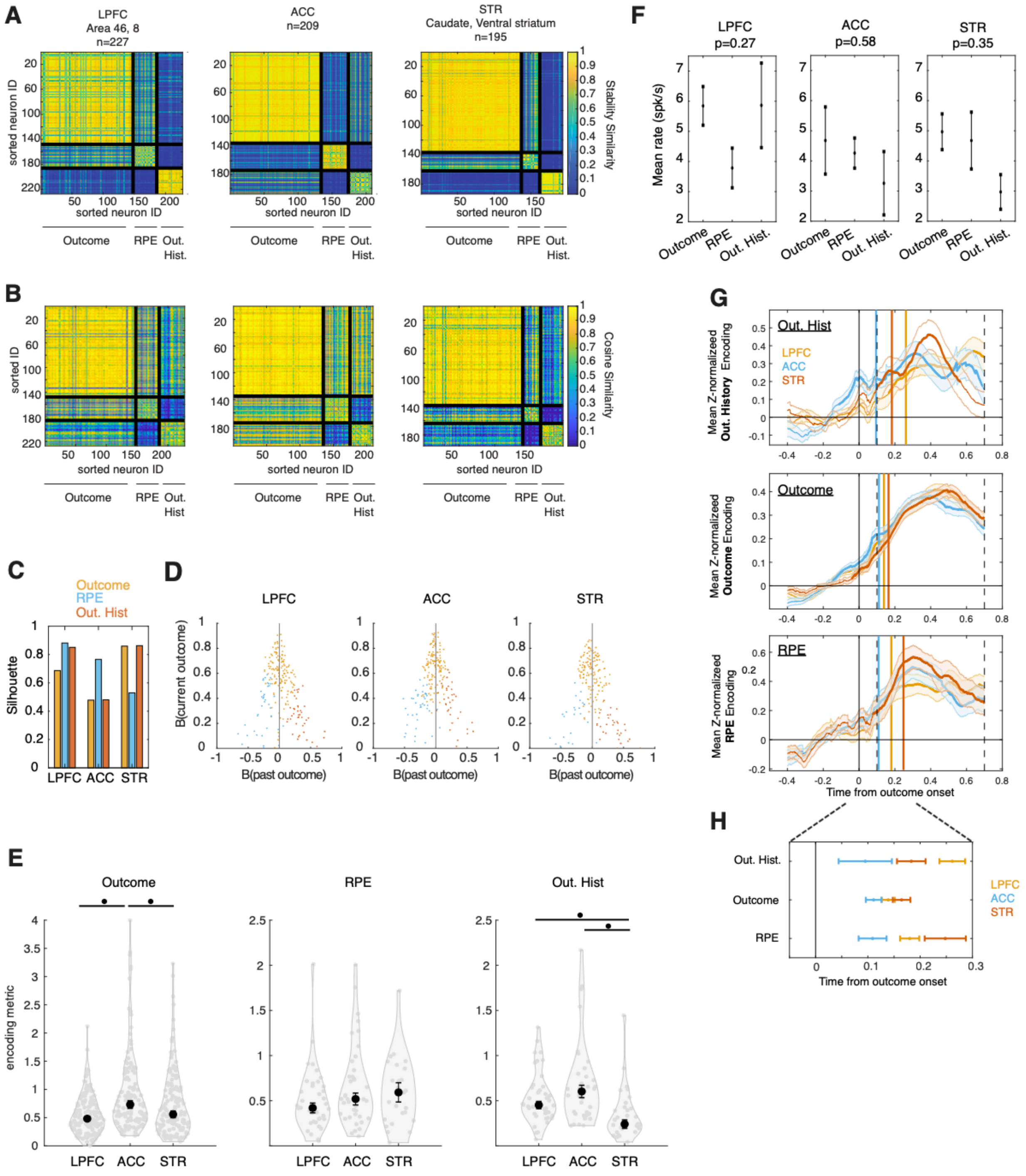

Supplementary Fig. 2. Assessing cluster characteristics

(**A**) Three encoding clusters emerge across the fronto-striatal axis. Pairwise similarity (pairwise stability) between pairs of neurons for each region. Neurons were sorted such that those in the same cluster were adjacent. (**B**) Same as (A) but using the cosine similarity. **(C)** Silhouette metric assessing differentiability of clusters, plotted separately for each area. **(D)** Normalized regression coefficients for previous (x-axis) and current (y-axis) outcome, for LPFC (left), ACC (middle), and STR (right). Points have been color coded by cluster assignment. Note that neurons where the

current errors were positively encoded (i.e. negative beta weight for current outcome), the sign for all regression weights was flipped (*see* **Methods**). **(E)** Violin plots depicting encoding strength for each encoding cluster, split by area. Black dots are the median, and vertical bars are the standard error of the median. Top/bottom 5% of values were trimmed for visualization purposes. **(F)** Median and standard error of firing rate of encoding clusters for each of three regions. Firing rate differences were similar within all clusters (Kruskal Wallis, $p>0.05$). (**G**) Time resolved, z-score normalized encoding metrics relative to the [-0.4 0] pre-outcome period, separated for *Outcome History* clusters (top), *Outcome* clusters (middle), and *RPE* clusters (bottom), and for LPFC (yellow), ACC (blue) and STR (red). At each time point, we assessed whether encoding is above the baseline period (Wilcoxon signrank test). The bolded lines represent the largest contiguous mass where encoding was above baseline. This region represents the time-of-interest over which the latency was calculated for each individual cell. Latency was defined as the point at which 10% area-under-the-curve for the TOI was reached. Vertical lines depict the median latency for each cluster. **(H)** Median and standard deviation of the encoding latency for each cluster of cells. All clusters showed significant encoding after the outcome onset.

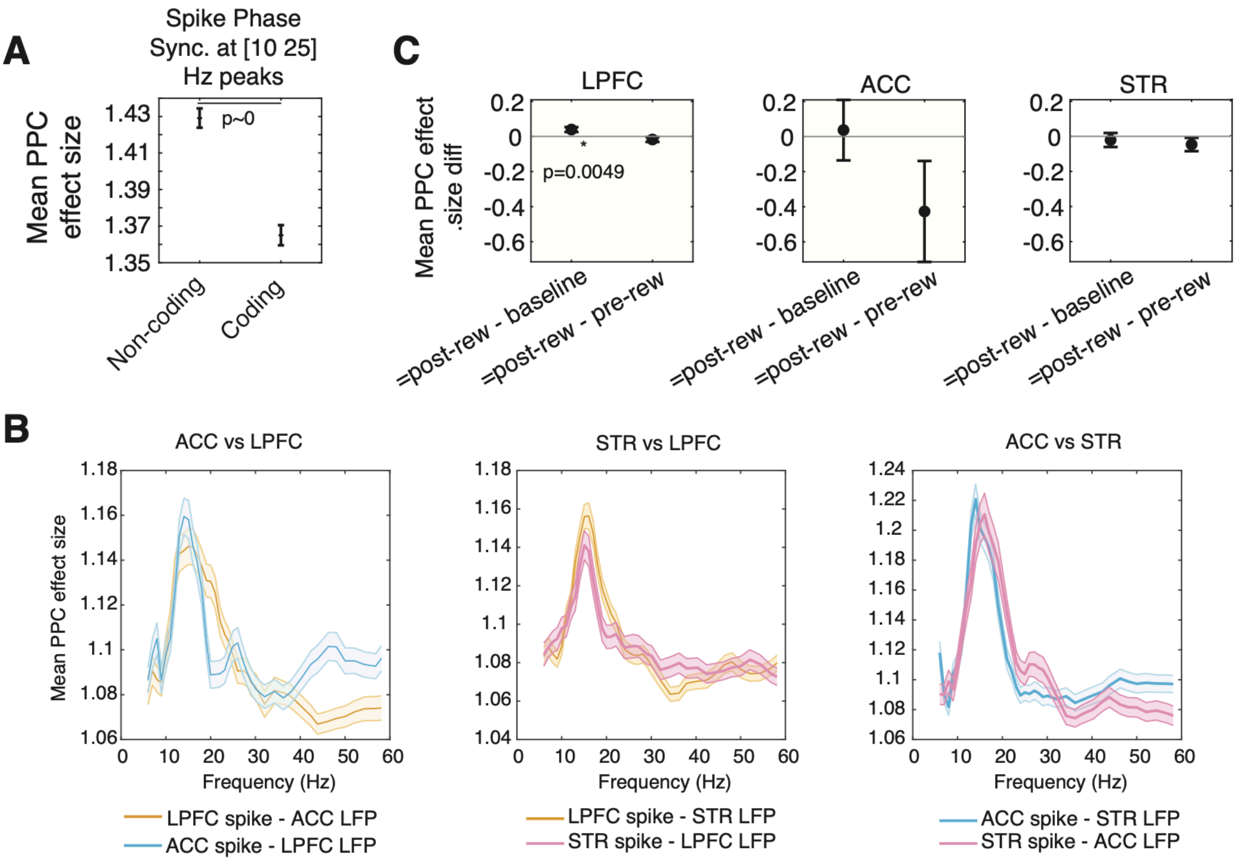

Supplementary Fig. 3. Inter-areal synchronization

(A) Mean and standard error of PPC effect size for prominent beta peaks for encoding and non-encoding pairs. Non-encoding pairs show significantly higher beta synchronization (ANOVA, p~0; N_{code}=3473, $n_{noncode}$=4465 (**B**) Mean and standard deviation of spike-LFP phase synchronization (PPC) between ACC and LPFC (left), STR and LPFC (middle) and between ACC and STR (right). (**C**) Median and standard error for difference in PPC effect, comparing either the post-reward period to a (pre-stimulus onset) baseline, or to a pre-reward period. Effects are plotted separately for each area. Paired T-test, two-sided

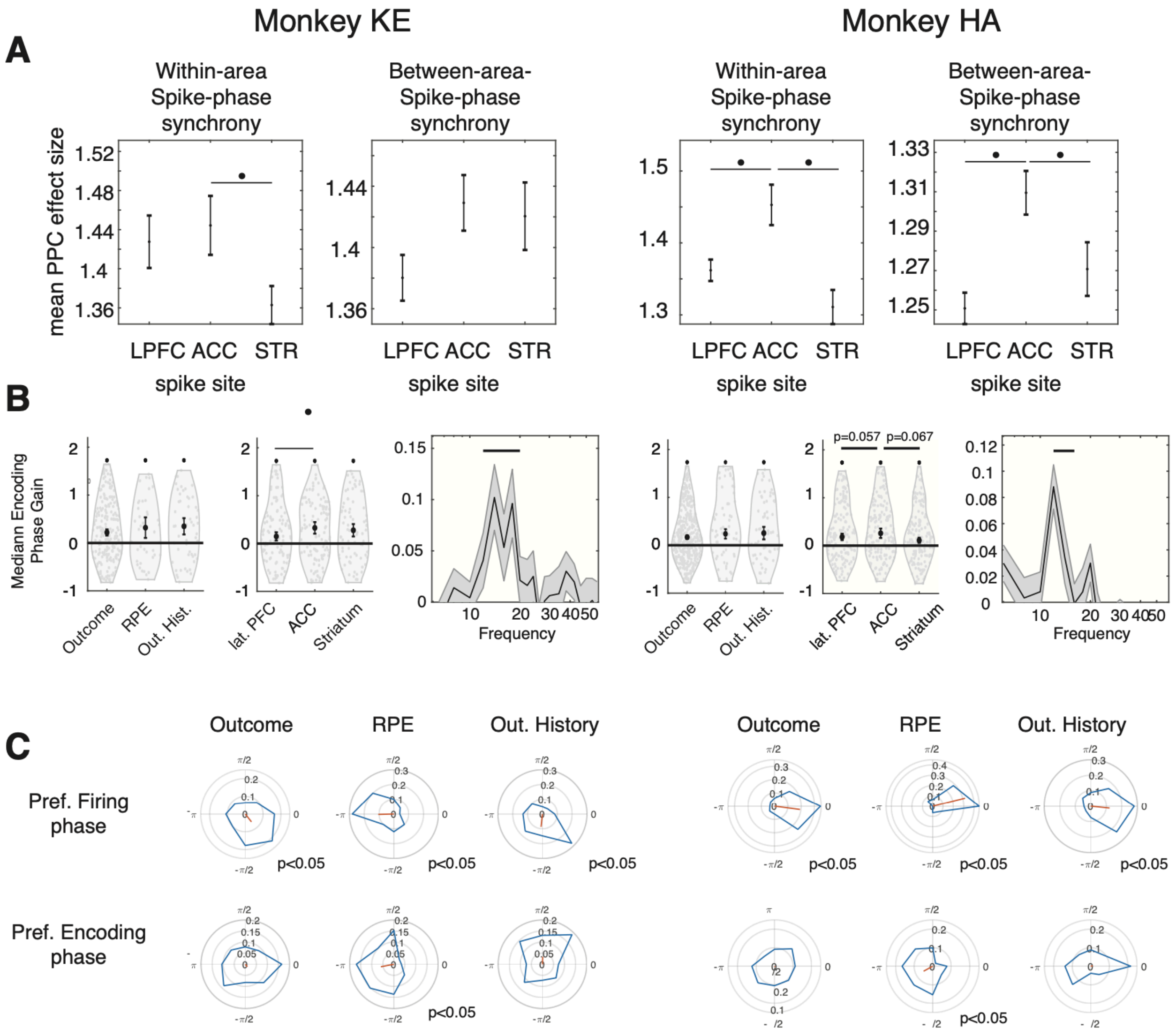

Supplementary Fig. 4. Summary of main results for individual monkeys

Individual main results for monkey KE (left) and HA (right). **(A)** Average spike-LFP phase synchronization for spike-LFP pairs within and between areas. (**B**) Average *EPFG* for each functional cluster (*left*), spike area *(middle*), and across frequencies (*right).* (**C**) Polar histograms of the preferred firing phase (upper panels) and maximal encoding phase (bottom panels) for each encoding cluster.

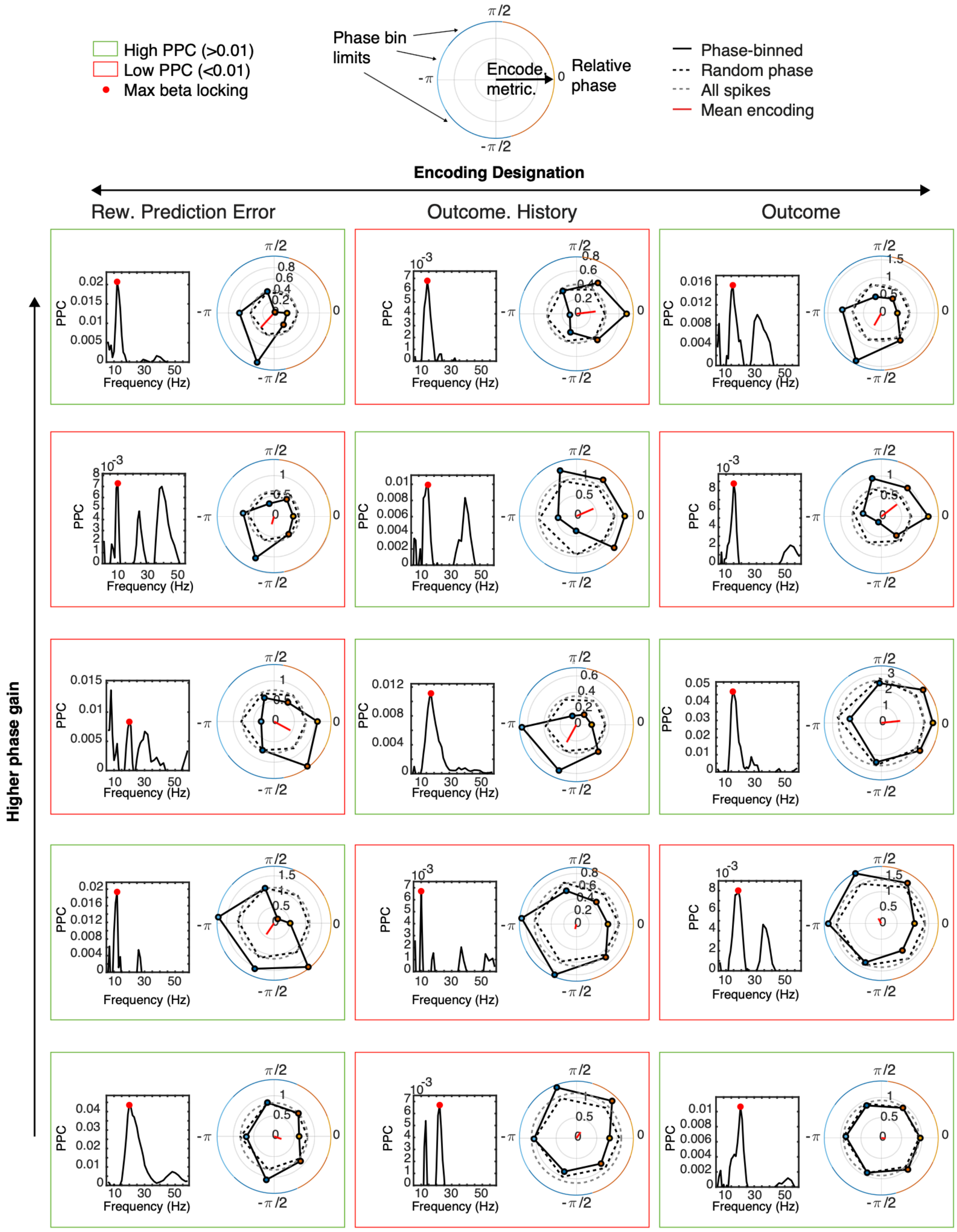

Supplementary Fig. 5. Encoding Phase Gain examples.
Columns depict the functional designation. Rows are ordered according to the relative phase gain, with lower phase gain at the bottom, and high phase gain at the top. The spike-phase consistency is depicted on the left, with the maximal significant locking in the [10 25] Hz beta band signified

with the red dot. The corresponding phase-dependent encoding is depicted on the right. Zero (0) corresponds to the preferred firing phase. Numbers on concentric circles are the value of the encoding metric. The grey dotted line represents the encoding metric estimated using all spikes, whereas the black dotted line represents the average across many spike-phase randomizations. The red line is the average direction. Colored border lines represent size of the bin. The colored box represents examples with a high (green) or low (red) degree of synchrony.

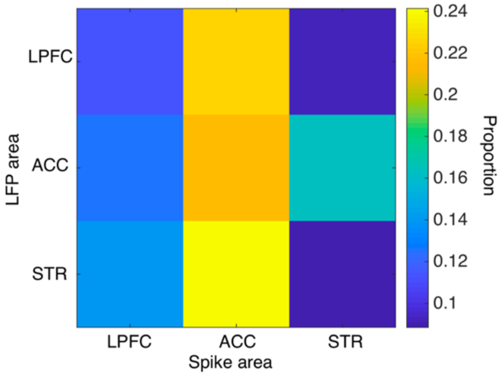

Supplementary Fig. 6. ACC has more spike-LFP pairs showing individually significant encoding phase gain

Proportion of spike-LFP pairs that exhibited individually significant *EPFG* (permutation test, $p<0.05$). The x-axis depicts the area from which spikes were taken, and the y-axis from where the LFP was taken. The proportion of encoding pairs was not distributed by chance (X^2 test, $p=0.0014$), with more significant pairs found in the ACC.

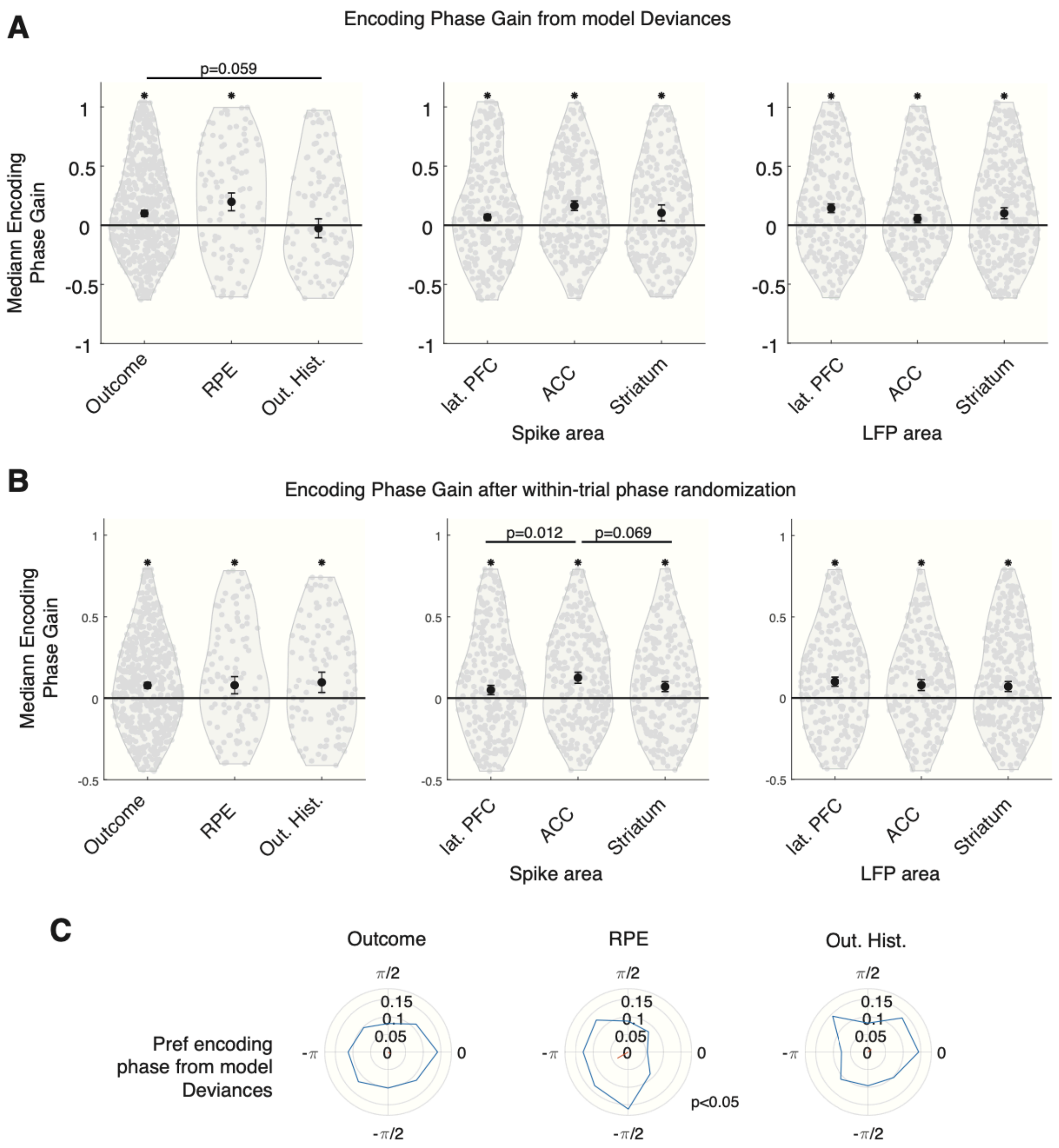

Supplementary Fig. 7. Alternative quantification of Encoding Phase-of-Firing Gain
Companion to Figure's 4 and 5 of the main text. **(A)** Companion to Fig. 4. Here, *EPFG* was determined by fitting a cosine to the Deviances[2], rather than the encoding metric from beta weights. Violin plots depicting encoding strength for each encoding cluster, split by encoding type (left), spike area (middle) and LFP area (right). Black dots are the median, and vertical bars are the standard error of the median. Top/bottom 5% of values were trimmed for visualization purposes. **(B)** Same format as (A) but encoding phase gain was calculated by randomizing phases within trials (instead of permutating them). **(C)** Companion to Fig. 5. Polar histograms of the preferred maximal encoding phase for each encoding cluster, derived from model deviances.

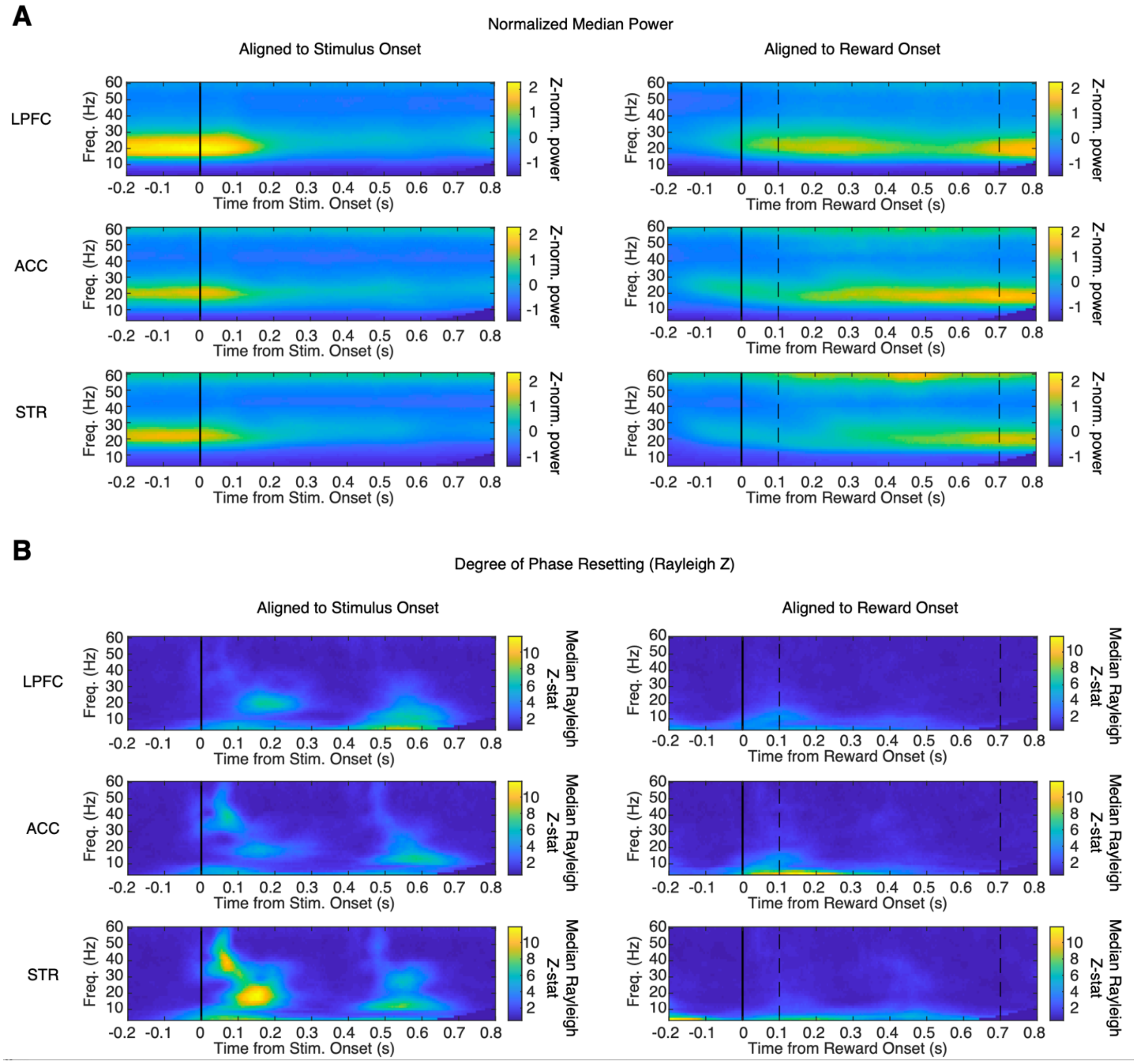

Supplementary Fig. 8. Beta power is prominent in the post-reward period
(A) Local field potential time-spectra plots detailing the median power in the LPFC (top), ACC (middle), and STR (bottom), aligned either to stimulus onset (left) or reward onset (right). Spectra were first normalized to account for 1/f structure of spectra. Each individual channel was then further z-score normalized across time and alignment (thus, left and right plots can be compared). **(B)** Median Rayleigh Z statistic assessing phase-resetting.

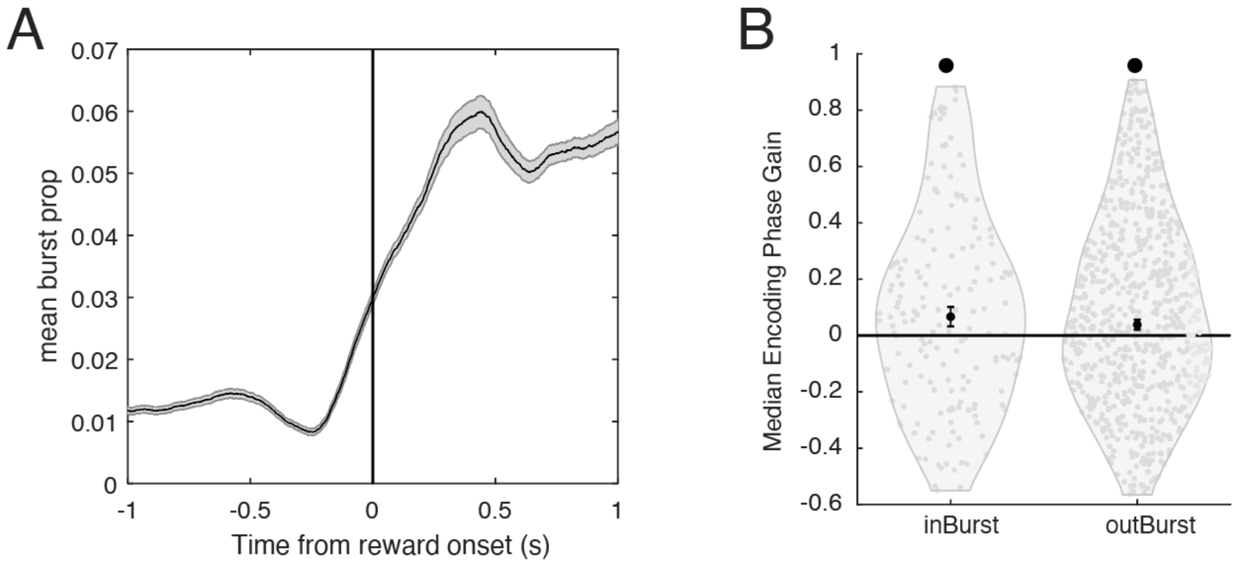

Supplementary Fig. 9. Phase-gain during beta bursting

(A) Probability of beta bursts aligned to reward onset. Bursts were defined as short periods where the average beta power exceeded 1.5 STD (*see* **Methods**). Beta bursts were more likely to occur post rather than pre-reward onset. **(B)** Phase gain assessed using spikes within (inBurst) or outside (outburst) burst periods. Although outBurst spikes showed a greater degree of phase gain, the difference was not significant.

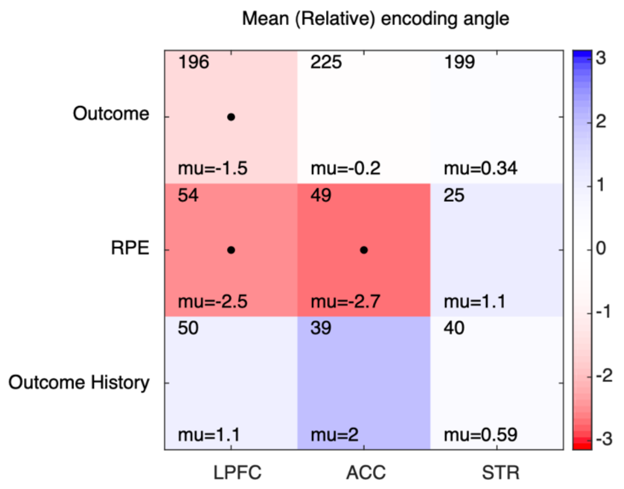

Supplementary Fig. 10. Preferred encoding for each area and function

Mean preferred encoding phase for *Outcome*, *RPE*, and *Outcome History* (y-axis) cells in the ACC, LPFC, and STR (x-axis). Color represents the (relative) encoding. Black dots represent significant phase concentration (Hodge-Ajne test, $p<0.05$). In each cell, the number of spike-LFP pairs that went into each cell is depicted in the top left, and the mean phase is on the bottom left. $P_{LPFC\text{-}outcome}=0.015$, $P_{LPFC\text{-}RPE}=0.011$, $P_{ACC\text{-}RPE}=0.001$

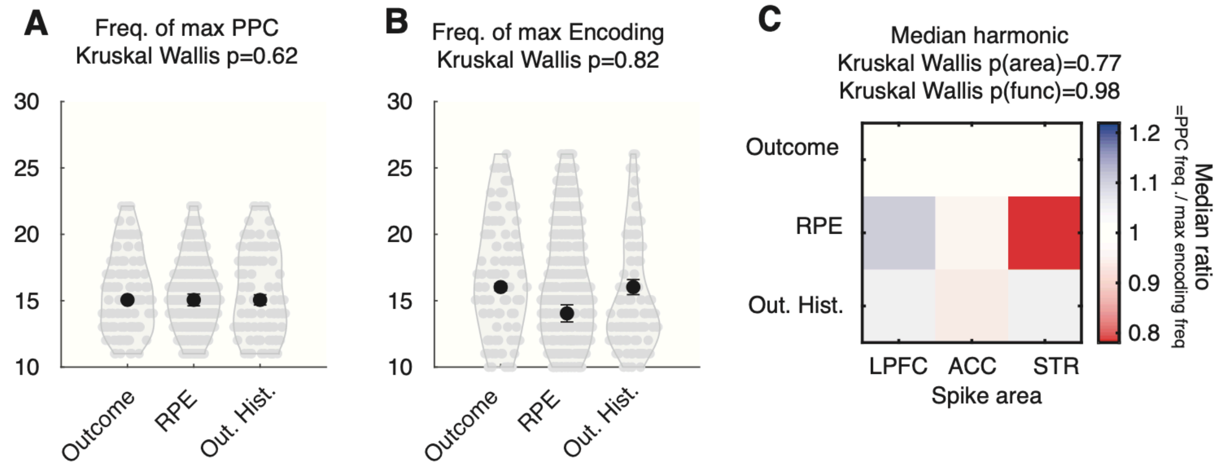

Supplementary Fig. 11. Frequency of maximal synchronization and encoding are matched **(A)** Median and standard errors of the frequency of the maximum PPC in the beta band for individual clusters. Top/bottom 5% of values were trimmed for visualization purposes. The overall median is ~15 Hz, with no differences between clusters. **(B)** Same as (A) but for the frequency exhibiting maximal encoding. No differences emerged between clusters. **(C)** Median ratio of the maximal synchronizing frequency and the maximal encoding frequency. The ratio did not vary as a function of area (Kruskal Wallis test, p=0.77) or encoding cluster (p=0.98). $n_{outcome}$=620, n_{RPE}=129, $n_{OutHist}$=128.
